# Treatment Behaviors of Patients With Odontogenic Infections During the COVID-19 Pandemic

**DOI:** 10.7759/cureus.81414

**Published:** 2025-03-29

**Authors:** Eiji Iwata, Yuriko Susukida, Junya Kusumoto, Akira Tachibana, Masaya Akashi

**Affiliations:** 1 Department of Oral and Maxillofacial Surgery, Kakogawa Central City Hospital, Kakogawa, JPN; 2 Department of Oral and Maxillofacial Surgery, Kobe University Graduate School of Medicine, Kobe, JPN; 3 Department of Oral and Maxillofacial Surgery, Graduate School of Medicine, Dentistry and Pharmaceutical Sciences, Okayama University, Okayama, JPN

**Keywords:** coronavirus disease 2019, deep neck infection, odontogenic infections, severity scale, treatment behavior

## Abstract

Objective: Using a severity scale, this study aimed to investigate the treatment behavior of patients with odontogenic infections during the COVID-19 pandemic.

Materials and methods:This study included patients admitted for odontogenic deep neck infection (DNI) treatment at a single center in Japan between 2017 and 2022. Participants were divided into two groups: a pre-COVID group before 2019 and a COVID group after 2020. Patient characteristics, clinical data, and DNI severity were compared.

Results: During the pandemic, the number of patients with mild DNIs decreased significantly, whereas the number of patients with severe DNIs did not change significantly, resulting in a significant increase in the latter proportion (42/101 (41.6%) vs. 26/43 (60.5%); p=0.045). In addition, the patients in the COVID group were significantly older than those in the pre-COVID group (median 66.0 years vs. 56.0 years; p=0.018).

Conclusions: Our results suggest that patients with odontogenic infections, especially the elderly, may have avoided hospitals until their symptoms became severe during the COVID-19 pandemic.

## Introduction

The novel COVID-19, caused by SARS-CoV-2, was first identified in Wuhan, China, in December 2019 and rapidly spread worldwide [1,2]. The first case of COVID-19 in Japan was reported in January 2020 [1]. Shortly after, on March 11, 2020, the World Health Organization declared COVID-19 a global pandemic [2]. Since SARS-CoV-2 is present in saliva, COVID-19 can be transmitted via aerosolized saliva [3,4]. Regular dental practices generate aerosols, making dental clinics high-risk sites for droplet and airborne infections [5]. Consequently, dental treatment was considered a high-risk activity, leading to a temporary decline in the activity index of dental clinics in Japan [6,7].

Untreated odontogenic infections can spread to deep neck spaces, resulting in deep neck infections (DNIs) if left untreated [8]. For instance, Pesis et al. reported a case in which untreated apical periodontitis progressed to deep neck abscess and required long intubation and a case in which untreated severe caries progressed to deep neck abscess and required thoracotomy [9]. Although their incidence has decreased with the use of antibiotics, DNIs cause substantial complications, including upper airway obstruction, mediastinitis, septic shock, and vascular thrombosis, leading to significant morbidity and mortality [10]. Among DNIs, deep neck abscesses and necrotizing soft tissue infections (NSTIs) are particularly severe, fatal, and referred to as severe DNIs [11]. Deep neck abscesses are pus collections that form within deep neck spaces, potentially obstructing the airways and spreading to critical areas such as the mediastinum, leading to life-threatening complications [12]. NSTIs, characterized by extensive tissue damage and systemic toxicity, represent the most severe and fatal forms of DNIs [13]. Therefore, we focused on DNI severity, including these severe DNIs, and investigated the treatment behavior of patients with odontogenic infections during the COVID-19 pandemic by using the severity scale. This survey may be useful not only for understanding the reality of aggravation of odontogenic infections during the COVID-19 pandemic but also for predicting countermeasures if a similar pandemic occurs.

## Materials and methods

Patients

This study included patients admitted for the treatment of odontogenic DNIs at Kakogawa Central City Hospital, Japan, between January 2017 and December 2022. Inclusion criteria were as follows: patients of both sexes, aged ≥18 years, who had been hospitalized for >48 hours and received intravenous antibiotics. Hospitalization criteria included clinical findings such as skin erythema, dysphagia, difficulty eating, and elevated inflammatory markers in blood tests. The exclusion criteria were as follows: patients hospitalized for personal reasons, including difficulty visiting the hospital, those with missing data critical to this study, and those who declined participation after the study's publication. Patients were divided into two groups: pre-COVID between 2017 and 2019 and COVID between 2020 and 2022.

Data collection

The following variables were retrospectively collected from the medical records: patient age, sex, compromised host, lesion of odontogenic cause (maxilla or mandible), location of odontogenic cause (anterior, premolar, or molar), fever when admitted, the extent of mouth opening, blood test data (C-reactive protein (CRP), white blood cell (WBC) count, and neutrophil-to-lymphocyte ratio (NLR)), DNI severity, intensive care unit (ICU) stay, and hospitalization period. All examinations, including radiographic imaging and blood tests, were performed at the time of admission. Patients were classified as immunocompromised if they had diabetes or kidney failure. DNI severity was graded into four categories, as described in a previous study [14]. Grade I indicated cellulitis; Grade II involved cellulitis with superficial abscess formation localized to a single region without deep anatomical space involvement; Grade III referred to profound abscess formation extending into the deep anatomical spaces and deep neck abscesses; and Grade IV was defined as NSTI. The definition of NSTI was based on previous studies [15,16]. The diagnosis was based on the diagnostic criteria by Fisher et al. [17] and Mathieu et al. [18] and confirmed by evidence of gas production on computed tomography (CT) images, intraoperative findings, and histopathology. While Grades III and IV are called severe DNIs, Grades I and II are called mild DNIs [11]. Therefore, contrast-enhanced CT images are obtained when grade III or IV disease is clinically suspected. Incisional drainage was performed urgently if an abscess had formed, and the drained pus was sent for bacterial culture. All CT images were acquired using a 64-slice CT system (Aquilion 64; Canon Medical Systems Corp., Tochigi, Japan) or a 128-slice CT system (SOMATOM Definition Flash, Siemens, Munich, Germany). The data were collected under standard head and neck CT scanning conditions (120 kV, 1-5 mm slice thickness) with automatic exposure control. Based on the CT scans, multiplanar reconstruction sections were prepared using Digital Imaging and Communications in Medicine (DICOM) viewer software (Zyostation2 Type H, Ziosoft Inc., Tokyo, Japan). The contrast agents used were Iomeron 300 (Eisai, Tokyo, Japan), Iopamirdol 370 (Hikari Pharmaceutical, Tokyo, Japan), and Omnipaque 300 (GE Healthcare, Chicago, IL, USA).

Ethical approval

This study adhered to the guidelines of the 1964 Declaration of Helsinki. Ethical approval was obtained from the Institutional Review Board of Kakogawa Central City Hospital (authorization number: 2024-38). The ethics committee approved this study and provided administrative permission to access the data used in this research. As this was a retrospective study, the research plan was published on the websites of the participating hospitals following the IRB's guidelines, allowing patients the opportunity to opt out.

Statistical analysis

All statistical analyses were performed using Ekuseru-Toukei 2016 software (Social Survey Research Information Co. Ltd., Tokyo, Japan). Comparison between the two groups was analyzed using the Mann-Whitney U test for ordinal variables and Fisher’s exact test or chi-square test for categorical variables. Statistical significance was set at p<0.05.

## Results

**Figure 1:**
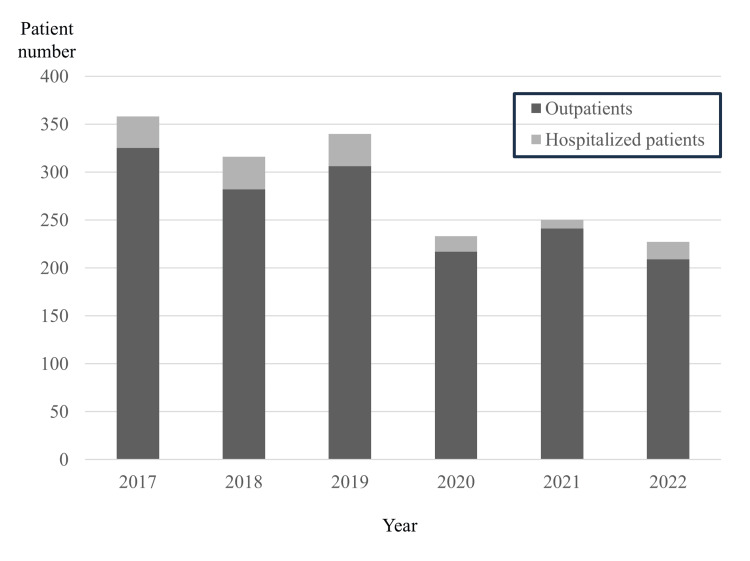
Distribution of outpatients and hospitalized patients by year

**Figure 2:**
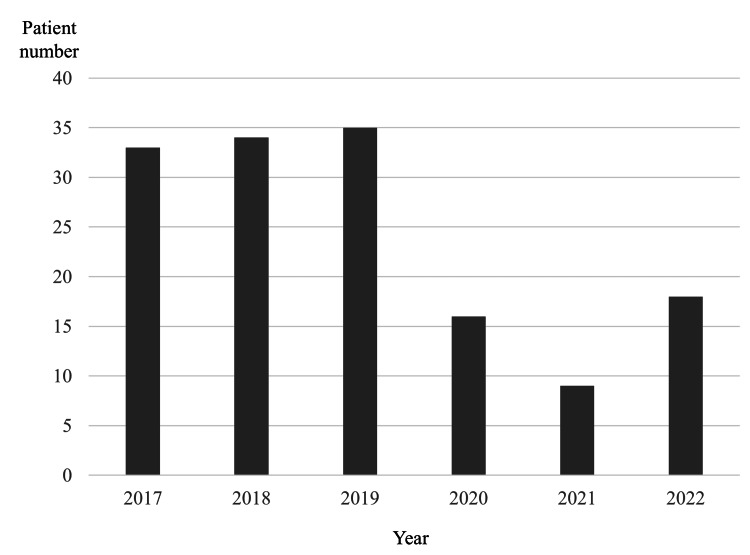
Changes in the number of hospitalized patients by year

Since 2020, the total number of patients with odontogenic infections and patients who needed hospitalization has decreased significantly (Figure 1). Among hospitalized patients, the pre-COVID group included 101 patients, whereas the COVID group included 43 (Figure 2).

**Figure 3:**
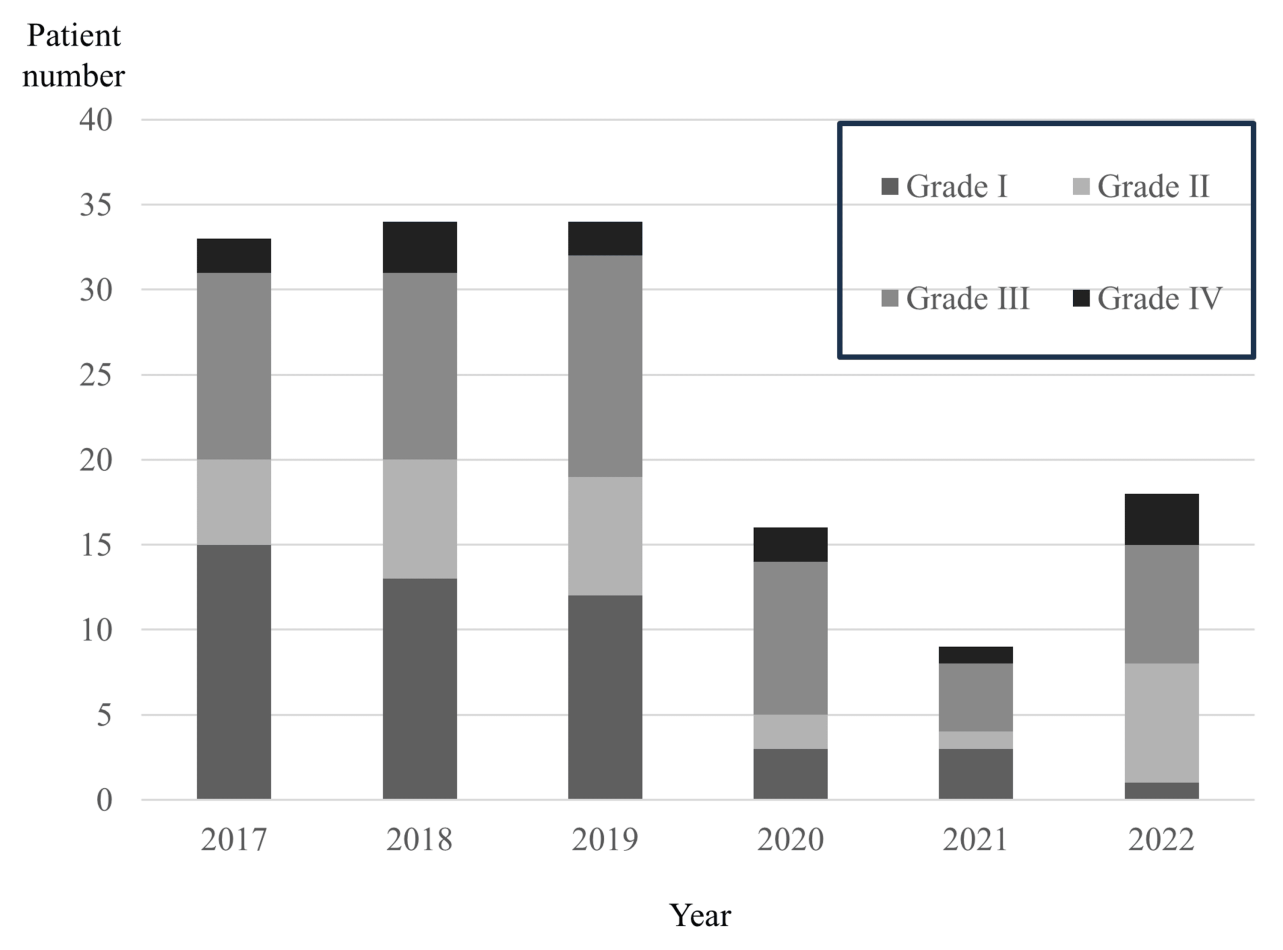
Distribution of DNI severity by year DNI: deep neck infection

Table 1 presents the results of comparisons between the two groups. The age of patients in the COVID group was significantly higher than in the pre-COVID group (median 66.0 years vs. 56.0 years; p=0.018). The number of compromised hosts was not significantly different between the two groups. In both groups, the mandibles and molars were the most common locations of odontogenic causes. In terms of symptoms, the COVID group had a higher fever and smaller mouth opening than the pre-COVID group, but the differences were not significant. In blood tests, CRP (median 15.0 mg/dL vs. 10.4 mg/dL) and NLR (median 8.4 vs. 6.5) were significantly higher in the COVID group than in the pre-COVID group (Table 1). In the pre-COVID group before 2019, the Grades I and II rates were over 50%, and those of Grades III and IV were <50% for each of the three years or even in total (Figures 3). Conversely, Grades I and II rates were <50%, and those of Grades III and IV were over 50% in the COVID group after 2020 for each of the three years or even in total (Figures 3). A significant difference was observed in the rate of severe DNI, including grades III and IV, between the two groups (42/101 (41.6%) vs. 26/43 (60.5%); p=0.045) (Table 1). However, during the pandemic, excluding 2021, the total number of patients decreased for some reason, and the number of patients with mild DNIs (i.e., Grades I and II) decreased significantly. In contrast, the number of patients with severe DNIs (i.e., Grades III and IV) did not change significantly (Figure 3). The number of patients admitted to the ICU was higher in the COVID group than in the pre-COVID group (8/43 (18.6%) vs. 9/101 (8.9%)). The median length of the hospitalization period was 9.0 days in both groups.

**Table 1:** Comparison of pre-COVID and COVID groups Values are presented as absolute numbers, with the corresponding percentages of the total in parentheses. The values in the right-hand column indicate the statistical significance of the differences between subgroups. Most variables are expressed as medians (range) on a non-parametric ratio scale. * p<0.05. COVID: coronavirus disease, CRP: C-reactive protein, WBC: white blood cell, NLR: neutrophil-to-lymphocyte ratio, DNI: deep neck infection, ICU: intensive care unit

Variable		Pre-COVID group (n=101)	COVID group (n=43)	p-value
Age (years)	Median (range)	56.0 (18–94)	66.0 (19–93)	0.018*
Sex	Male	51 (50.5%)	22 (51.2%)	1.000
	Female	50 (49.5%)	21 (48.8%)	
Compromised host	Yes	30 (29.7%)	18 (41.9%)	0.179
	No	71 (70.3%)	25 (58.1%)	
Lesion	Maxilla	11 (10.9%)	7 (16.3%)	0.413
	Mandible	90 (89.1%)	36 (83.7%)	
Location of odontogenic cause	Anterior	12 (11.9%)	9 (20.9%)	0.351
	Premolar	11 (10.9%)	5 (11.6%)	
	Molar	78 (77.2%)	29 (67.5%)	
Fever (℃)	Median (range)	37.0 (36.0–40.0)	37.2 (35.8–39.9)	0.835
Extent of opening the mouth	Median (range)	30.0 (0–50)	20.0 (0–50)	0.349
CRP (mg/dL)	Median (range)	10.4 (0.5–43.4)	15.0 (1.4–39.7)	0.007*
WBC (10^3^/µL)	Median (range)	12.4 (3.8–33.5)	11.9 (4.0–21.0)	0.586
NLR	Median (range)	6.5 (1.8–38.9)	8.4 (2.3–97.0)	0.042*
DNI severity	Severe DNIs (Grade III/IV)	42 (41.6%)	26 (60.5%)	0.045*
	Mild DNIs (Grade I/II)	59 (58.4%)	17 (39.5%)	
ICU stay	Yes	9 (8.9%)	8 (18.6%)	0.155
	No	92 (91.1%)	35 (81.4%)	
Hospitalization period (days)	Median (range)	9.0 (3–60)	9.0 (5–108)	0.870

Table 2 shows the results of causative bacteria. In both groups, the proportion of obligate anaerobes was higher than that of facultative anaerobes, and this tendency was stronger in the COVID group than in the pre-COVID group (32/16 vs. 42/35). Among the facultative anaerobic bacteria, *Streptococcus constellatus *was the most common in both the pre-COVID and COVID groups. *Parvimonas micra* was the most common among the obligate anaerobic bacteria, followed by *Prevotella* spp. All patients underwent empirical treatment with intravenous antibiotics for both gram-positive and gram-negative bacteria. This was adjusted as necessary after the final culture and sensitivity results were obtained by microbiology. Sulbactam/ampicillin was the most commonly used antibiotic (data not shown). The other antibiotics used included clindamycin, ceftriaxone, and meropenem (data not shown).

**Table 2:** Distribution of microorganisms as causative bacteria (a) *Streptococcus constellatus:* 11 (31.4%); *Streptococcus anginosus:* 8 (22.9%); *Streptococcus​​​​​​​ intermedius:* 6 (17.1%); *Streptococcus​​​​​​​ cristasus:* 1 (2.9%); *Streptococcus​​​​​​​ gordonii: *1 (2.9%); *Streptococcus*​​​​​​​* mitis:* 1 (2.9%); *Streptococcus​​​​​​​ oralis: *1 (2.9%); *Streptococcus​​​​​​​ salivarus:* 1 (2.9%); not identified: 1 (2.9%) (b) *Streptococcus​​​​​​​ constellatus: *5 (31.3%); *Streptococcus​​​​​​​ anginosus:* 4 (25.0%); *Streptococcus​​​​​​​ intermedius:* 2 (12.5%); *Streptococcus​​​​​​​ gordonii:* 1 (6.3%); *Streptococcus​​​​​​​ sanguinis:* 1 (6.3%) COVID: coronavirus disease

Pre-COVID group (n=32/61)	No.	COVID group (n=28/36)	No.
Facultative anaerobic bacteria	35 (100.0%)	Facultative anaerobic bacteria	16 (100.0%)
*Streptococcus *spp. ^a^	31 (88.5%)	*Streptococcus *spp. ^b^	14 (87.5%)
*Actinomyces* spp.	2 (5.7%)	*Propinonibacterium* spp.	2 (12.5%)
*Staphylococcus* spp.	1 (2.9%)	-	-
Enterobacter cloacae	1 (2.9%)	-	-
Obligate anaerobic bacteria	42 (100.0%)	Obligate anaerobic bacteria	32 (100.0%)
Parvimonas micra	15 (35.7%)	Parvimonas micra	13 (40.6%)
*Prevotella* spp.	10 (23.8%)	*Prevotella *spp.	12 (37.5%)
*Peptostreptococcus* spp.	6 (14.3%)	*Peptostreptococcus* spp.	2 (6.3%)
*Porphyromonas *spp.	5 (11.8%)	Fusobacterium nucleatum	2 (6.3%)
Fusobacterium nucleatum	2 (4.8%)	*Anaelococcus* spp.	1 (3.1%)
Anaelococcus spp.	2 (4.8%)	*Veillonella *spp.	1 (3.1%)
*Veillonella *spp.	1 (2.4%)	Finegoldia magna	1 (3.1%)
Finegoldia magna	1 (2.4%)	-	-

## Discussion

Using a severity scale, this study investigated the treatment behavior of patients with odontogenic infections during the COVID-19 pandemic. During the pandemic, the number of patients with mild DNIs decreased significantly. In contrast, the number of patients with severe DNIs did not change significantly, resulting in a significant increase in the latter proportion (42/101 (41.6%) vs. 26/43 (60.5%); p=0.045). In addition, the patients in the COVID group were significantly older than those in the pre-COVID group. Although the proportion of patients admitted to the ICU was higher in the COVID group than in the pre-COVID group (8/43 (18.6%) vs. 9/101 (8.9%)), the median length of hospitalization was the same in both groups.

We found that the number of patients with mild DNIs decreased significantly, whereas the number of patients with severe DNIs did not change significantly during the pandemic. This suggests that patients with mild DNI may have avoided visiting hospitals. As a result, the rate of patients with severe DNIs was significantly higher in the COVID group than in the pre-COVID group. The patients in the COVID group also had significantly elevated CRP and NLR levels. The liver produces CRP, a non-specific acute-phase protein, in response to pro-inflammatory cytokines such as interleukin-6 [19]. Ho et al. reported that elevated CRP levels in DNIs indicate significant inflammation and correlate with the infection severity [20]. The NLR is a marker of systemic inflammation [21]. An elevated NLR signifies a heightened inflammatory response [21,22]. Previous studies have shown that a higher NLR in the DNIs may indicate more severe or extensive infection [22]. Regarding causative bacteria, this study found that *Streptococcus* spp. was predominant among the facultative anaerobes. In contrast, *Prevotella* spp. and *Pavimonas micra* were predominant among the obligate anaerobes, similar to previous studies [23,24]. The proportion of obligate anaerobes was higher than that of facultative anaerobes in both groups, and this tendency was stronger in the COVID group than in the pre-COVID group (32/16 (200%) vs. 42/35 (120%)). Generally, obligate anaerobic bacteria are known to be deeply involved in the DNI severity [25,26]. These results suggest that the patients in the COVID group had more severe odontogenic infections than those in the pre-COVID group. This result was similar to previous studies [27,28]. Only two studies showed the severity of odontogenic infections during the COVID-19 pandemic [27,28]. Louizakis et al. investigated 341 Greek patients with severe odontogenic infections who required hospitalization before and during COVID-19 and reported an increased number of patients with COVID-19 who needed ICU admission (23 patients vs. 3 patients) [27]. Grill et al. investigated 961 German patients with odontogenic or intraoral abscesses before and during COVID-19 and reported a significantly higher number of patients with advanced abscesses requiring intervention under general anesthesia [28]. The number of patients who required ICU stay and the length of the hospitalization period were not significantly different in this study before and during the COVID-19 pandemic. This suggests that we responded quickly during the COVID-19 pandemic, just as we did before the COVID-19 pandemic.

We also found that patients in the COVID group were significantly older than those in the pre-COVID group. Although older age is a risk factor for DNI severity [15,29], the higher age in the COVID group also may reflect changes in patients’ behavior during the COVID-19 pandemic. Several studies have shown that the COVID-19 pandemic significantly declined emergency department visits across various medical disciplines [30,31]. Fear of SARS-CoV-2 infection likely led many patients to avoid hospitals [30,31]. This avoidance was particularly notable among older adults who, due to their weakened immune systems, faced higher risks of severe complications if infected. A recent study reported that the total number of older adult patients with emergency department visits to many hospitals dropped considerably during the pandemic [32]. Despite that, we found that patients in the COVID group were significantly older than those in the pre-COVID group. This suggests that patients with odontogenic infections, especially the elderly, may have avoided hospitals until their symptoms became severe during the COVID-19 pandemic.

COVID-19 spreads through direct transmission (e.g., coughing, sneezing, and inhaling droplets) and contact transmission (e.g., touching the oral, nasal, and eye mucous membranes with contaminated surfaces, droplets, and aerosols). Dental practices use rotary dental and surgical tools, including handpieces, ultrasonic scalers, and air-water syringes, which produce visible sprays containing water droplets, saliva, blood, microorganisms, and other debris. Therefore, dental clinics are high-risk locations for the spread of COVID-19 infection between patients and healthcare providers [33,34]. This caused many patients to fear the risk of contracting COVID-19, resulting in postponing or canceling dental visits [35]. Several studies reported lower dental care demands during the COVID-19 pandemic in different parts of the world, including Japan [7,36,37]. In response, many dental clinics and oral surgery departments of hospitals in Japan proactively implemented standard precautions [37,38]. To our knowledge, only one cluster outbreak has been reported in a dental clinic in Japan [38]. Tanaka et al. investigated 51 Japanese faculty members belonging to the Department of Oral and Maxillofacial Surgery in university hospitals and reported that all members proactively implemented standard precautions, and 14 faculty members treated patients with COVID-19; however, no infections were transmitted from the patients to the medical staff [39]. They also reported that several patients were found to have an infection after treatment (i.e., medical staff came into close contact). Still, no transmission from patients to medical staff was observed, and it was concluded that COVID-19 clusters are unlikely to occur in dental and oral surgical care settings if appropriate protective measures are implemented [39]. In this study, with proactive standard precautions, no in-hospital clusters were observed for any procedure, including drainage surgery under general anesthesia (data not shown). These results suggest that in the event of a similar pandemic in the future, patients with odontogenic infections can comfortably visit a dental clinic and receive dental and oral surgical care in hospitals, even if their condition becomes severe.

To our knowledge, this is the first reported study to investigate the treatment behavior of patients with odontogenic infections during the COVID-19 pandemic by using a severity scale and the first to investigate odontogenic DNIs in Japan during the pandemic. However, this study has some limitations. First, as this was a retrospective study, unknown confounding factors are possible. Second, the sample size and number of outcomes evaluated in this study were small, which could have introduced bias in data selection and analysis. However, severe odontogenic DNIs and pandemics are rare. We could not compare the two groups using matching conditions. Finally, the treatment content, including the use of antibacterial drugs, inevitably influences the prognosis, including the length of hospitalization. Whether antibiotics were administered before hospitalization remains unknown. We should accept our results based on this understanding.

## Conclusions

Our results suggest that patients with mild DNI may have avoided visiting hospitals. In addition, despite elderly people tending to avoid hospitals for fear of COVID-19 infection, the patients in the COVID group were significantly older than those in the pre-COVID group in this study. These suggest that patients with odontogenic infections, especially the elderly, may have avoided hospitals until their symptoms became severe during the COVID-19 pandemic. However, only one cluster has been reported in a dental clinic in Japan due to proactively implemented standard precautions. In previous Japanese studies and this study, no in-hospital clusters were observed for dental and oral surgical care in hospitals. Our results suggest that in the event of a similar pandemic in the future, patients with odontogenic infections can comfortably visit a dental clinic and receive dental and oral surgical care in hospitals, even if their condition becomes severe. However, as nothing beats early detection and therapeutic interventions, regular oral health management in dental clinics is important.
